# Diagnostic Value of Serum Procalcitonin and Erythrocyte-bound Complement Component 4d (E-C4d) Levels in Differentiating a Flare-up from a Bacterial Infection in Egyptian Patients with Systemic Lupus Erythematosus

**DOI:** 10.7759/cureus.100460

**Published:** 2025-12-30

**Authors:** Mona A Abd El-Hamid, Niveen A Elwakeel, Medhat M Ali, Sherif R El-Basiouny, Ghada M Elnady

**Affiliations:** 1 Medical Microbiology and Immunology, Faculty of Medicine, Mansoura University, Mansoura, EGY; 2 Rheumatology and Rehabilitation, Faculty of Medicine, Mansoura University, Mansoura, EGY; 3 Pathological Sciences, Fakeeh College for Medical Sciences, Jeddah, SAU

**Keywords:** bacterial infection, e-c4d, erythrocyte-bound complement component 4d (e-c4d), flare, procalcitonin, systemic lupus erythematosus

## Abstract

Background

The distinction between bacterial infection and febrile flare in patients with systemic lupus erythematosus (SLE) has always been a crucial yet difficult step in the management of these patients. Although the presentation looks similar, the treatment is extremely different and increases the burden on health care systems, particularly in low-income countries.

Aim of this study

This study aimed to investigate the potential value of combining serum procalcitonin (PCT) and erythrocyte-bound Complement Component 4d (E-C4d) measurements in distinguishing infection from disease flare in patients with SLE.

Methods

Serum levels of these two biomarkers were analyzed in patients with positive aerobic bacterial cultures, patients with flare-up fever showing no signs of infection, and a control group with medical diseases other than SLE.

Results

The PCT level was found to be high in patients with bacterial infections and is considered a suitable marker to exclude cases of SLE with bacterial infections. Additionally, E-C4d was found to have a positive correlation with SLE flare-ups.

Conclusion

Our findings suggest that the use of serum PCT and E-C4d simultaneously may help differentiate between lupus flares and bacterial infection. However, this observation needs further validation in larger studies.

## Introduction

Systemic lupus erythematosus (SLE) is defined as an autoimmune disease that is more common in females, particularly those aged 15-40 years, and is linked to severe inflammation in all body systems [1]. Infections have been found to be the leading cause of death in both high-income and low-/middle-income countries [2], with bacterial infections reported in up to 75% of cases [3]. The high likelihood of infections in these patients is attributed to immunosuppressant therapy [4].

Differentiation of bacterial infections from flares in SLE patients is quite challenging, as the clinical presentations of both conditions are similar [5]. However, the treatment of bacterial infections is quite different from that of lupus flares; hence, a correct diagnosis becomes of utmost importance in the decision-making of treatment approaches [6]. Unfortunately, traditional laboratory screening tests, including leukocyte numbers, the presence of immature leukocytes, the C-reactive protein (CRP) level, and the erythrocyte sedimentation rate (ESR), which are used for the diagnosis of infections, have reduced sensitivity and specificity. Although culture results are often confirmatory, they are rarely used to guide treatment decisions due to their relatively long turnaround times, which can exceed 72 hours [7]. On the other hand, anti-double-strand DNA (anti-dsDNA) antibodies, in addition to the serum complement proteins C3 and C4, are used for the assessment of SLE activity. Nevertheless, the greatest number of SLE patients present continuously high levels of anti-dsDNA antibodies or reduced levels of complement components C3 and C4. Hence, there is a critical need to explore alternative, rapid biomarkers that offer superior discriminatory value between SLE flare and bacterial infection [8].

Procalcitonin (PCT) can be considered a good marker for discriminating infections from flares in SLE patients. Its serum levels are usually increased in patients with bacterial infections [9]. The serum PCT level is more specific than the CRP and leukocyte levels for inflammation caused by bacterial and mycotic infections, and the serum PCT level is not increased or slightly increased in patients with autoimmune diseases [10].

Another biomarker of differentiation is the proteolytic fragment of C4d, which is present on the surface of normal erythrocytes (erythrocyte-bound complement component 4d (E-C4d)). Lupus flares have been found to be associated with E-C4d, as shown by several studies [11]. This distinct association positions E-C4d as a promising biomarker for disease activity, raising the possibility that its combined use may help distinguish flare from infection.

Given these challenges, this study aims to evaluate the diagnostic value of serum PCT and E-C4d levels in distinguishing between an SLE flare and a bacterial infection in Egyptian SLE patients, thereby contributing to timely clinical decision-making.

An earlier version of this work was presented as a poster at the 5^th^ European Congress on Infectious Diseases, held on October 9 to 10, 2023, in London, UK.

## Materials and methods

This cross-sectional study was conducted over a period of 24 months from November 2022 to November 2024. The study included subjects attending Mansoura University outpatient clinics and hospitals in Mansoura, Egypt. A convenience sample of N=80 patients and control subjects was recruited. This study was approved by the institutional review board and research ethics committee of Mansoura University (approval number: MD/16.06.31).

All consecutive patients meeting the criteria for each group were enrolled until 20 patients were included in each. The first group included SLE patients with lupus activity, and the second included 20 SLE patients without evidence of activity but with diagnosed bacterial infection as proven by positive culture. The third group included 20 SLE patients who attended outpatient clinics for follow-up and had no evidence of flares or infections. The fourth group included 20 non-SLE subjects presenting with fever in which bacterial infections were culture-proven. Exclusion criteria included (1) failure to meet the diagnostic criteria required for assignment to any of the study groups; (2) the presence of any acute inflammatory condition at the time of sampling, such as active infection, recent trauma, or postoperative inflammatory response; (3) chronic inflammatory diseases, including but not limited to chronic liver disease, chronic kidney disease, or inflammatory bowel disease; and (4) any diagnosed autoimmune disorder (e.g., autoimmune thyroiditis or rheumatoid arthritis) that could independently influence serum PCT levels or confound the interpretation of inflammatory biomarkers.

SLE was diagnosed on the basis of both clinical and laboratory findings, including inflammatory markers, autoantibodies, complement levels, and urinalysis, according to the updated criteria for the sorting of SLE [12]. Disease flares were defined according to the Systemic Lupus Erythematosus Disease Activity Index (SLEDAI), with a score of 3 or more on admission [13]. Infection was defined on the basis of clinical symptoms, further explained by microbiological isolation from the suggested site of infection and clinical improvement following antimicrobial therapy.

Representative samples were obtained for culture and sensitivity testing. In addition, peripheral blood samples were collected before the initiation of treatment of lupus activity or infection. Evaluation of E-C4d levels was done using flow cytometry. Erythrocytes were stained with a fluorochrome-conjugated anti-C4d antibody (Abcam, Cambridge, UK). RBCs were identified by their characteristic forward and side scatter properties before analyzing fluorescence intensity using standard instrument settings. Single-stained compensation controls and fluorescence-minus-one (FMO) controls were included for accurate gating. Data were gated to exclude debris and doublets. Flow cytometry acquisition was performed on a BD Biosciences instrument (San Jose, CA, USA), and data were analyzed using logicle transformation. Outcomes included the percentage of C4d-positive erythrocytes.

Serum PCT levels were measured via a human PCT enzyme-linked immunosorbent assay (ELISA) kit (Bioassay Technology Laboratory, Shanghai, China) according to the manufacturer’s instructions.

All serum samples were coded and analyzed by laboratory staff who were blinded to the clinical groups. Data analysis was performed by an independent analyst who had no access to group identifiers.

Statistical analysis

The data were entered and statistically analyzed via the IBM SPSS Statistics software, version 21 (IBM Corp., Armonk, NY, USA). Qualitative data were presented as numbers and percentages. The chi-square ( χ²) test or Fisher's exact test was used for comparisons between groups, as appropriate. The quantitative data were presented as the means or medians, as appropriate. The data were tested for normality via the Kolmogorov‒Smirnov test. For normally distributed variables, an independent sample t-test was used for comparisons between groups, whereas for nonnormally distributed variables, the Mann‒Whitney test was used for comparisons between groups, and the Wilcoxon signed rank test was used for comparisons within groups. Pairwise tests were conducted for prespecified comparisons to evaluate differences between groups.

Receiver operating characteristic (ROC) curves were plotted. The area under the ROC curve (AUC) was calculated to describe the predictive accuracy and determine the cutoff point for the most sensitive parameters. A p-value < 0.05 was considered statistically significant, whereas a p-value < 0.001 was considered highly significant.

## Results

Demographic characteristics of the patients are summarized in Table 1. The table presents the age and sex of the study groups.

**Table 1:** Demographic data of the patients SD: standard deviation; SLE: systemic lupus erythematosus; p1: SLE flare-up group vs. SLE without bacterial infection or activity; p2: bacterial infection in SLE patients group vs. SLE patients without bacterial infection or activity; p3: bacterial infection in SLE patients group vs. lupus flare up group; p4: bacterial infection in SLE patients group vs. non-SLE patients with verified bacterial infection group.

Demographic data	SLE flare-up group (N=20)	Bacterial infection in SLE patients (N=.20)	SLE patients without activity or bacterial infections (N=20)	Non-SLE patients with verified bacterial infection (N=20)	p-value
Age (Years, Mean ± SD	30.90 ± 11.823	34.45 ± 10.625	33.00 ± 7.780	45.25 ± 21.918	p1=0.512, p2=0.625, p3=0.324, p4=0.055
Sex (Female/Male)	20 (100.0%) 0 (%)	20 (100%) 0 (0%)	19 (95.0) 1 (5.0)	14 (70.0) (30.0	p1=0.324, p2=0.324, p 3=0.830, p4=0.001

PCT diagnostic performance

A comparison of the four study groups revealed significantly greater serum PCT levels in the infection groups than in the non-infection group and the control group, as shown in Table 2.

**Table 2:** Results of PCT measurement PCT: procalcitonin; SLE: systemic lupus erythematosus; p1: SLE flare-up group vs. SLE without bacterial infection or activity; p2: bacterial infection in the SLE patient group vs. SLE patients without bacterial infection or activity; p3: bacterial infection in the SLE patient group vs. the lupus flare-up group; p4: bacterial infection in the SLE patient group vs. the non-SLE patients with verified bacterial infection group.

PCT (pg/ml)	SLE flare-up group (N= 20)	Bacterial infection in SLE patients (N= 20)	Non-SLE patients with culture-proven bacterial infection (N=20)	SLE patients without activity or bacterial infections (N= 20)	p-value
Mean ± SD	198.280± 83.581	536.396± 251.847	670.920± 321.127	210.355± 83.629	p1=0.662
					p2=<0.001
					p3=<0.001
					p4=0.149

**Figure 1:**
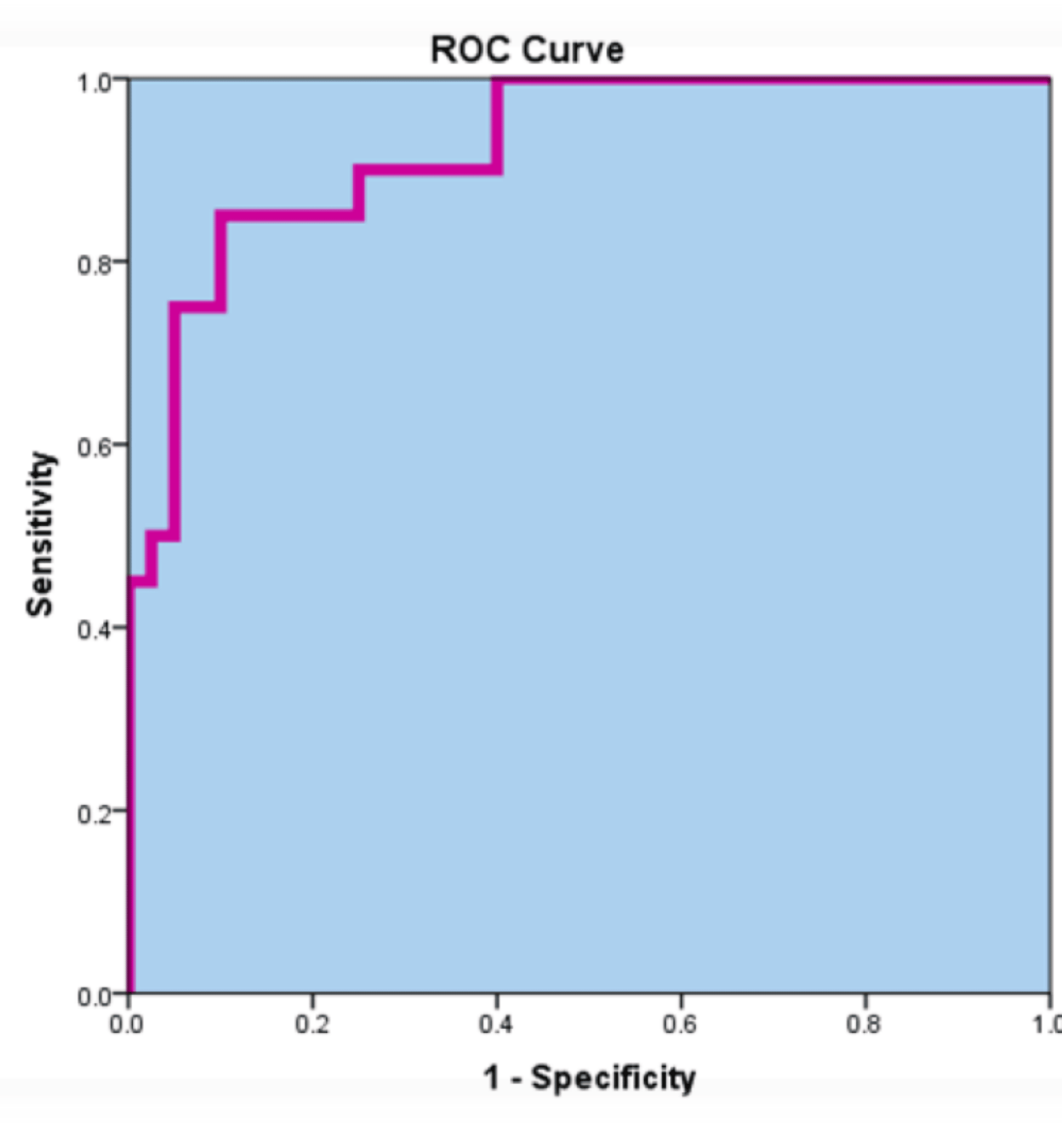
ROC curves comparing sensitivity and specificity of PCT for the diagnosis of bacterial infection in SLE The cutoff value for PCT was 298.66 pg/ml (0.299 ng/ml), at which sensitivity (85%) and specificity (90%) had the best combination, while the PPV and NPV were 87.8% and 95%. PCT: procalcitonin; ROC: receiver operating characteristic; SLE: systemic lupus erythematosus; PPV: positive predictive value; NPV: negative predictive value

An ROC curve was plotted to determine a cutoff value of PCT to diagnose bacterial infections in those patients. The sensitivity was 85% (95% CI: 76-92%) and specificity 90% (95% CI: 83-95%) of the best combination were 298.66 pg/ml (0.299 ng/ml), while the positive predictive value (PPV) and negative predictive value (NPV) were 87.8% (95% CI: 79-93%) and 95% (95% CI: 90-99%), respectively (Figure 1).

Role of E-C4d in activity differentiation

The levels of serum E-C4d were measured via flow cytometry (fluorescence intensity) and analyzed. Compared with those in other groups with or without infection, these parameters were found to be significantly elevated in SLE patients with a flare-up (p3≤0.001) (Table 3). Furthermore, these levels were positively correlated with SLEDAI lupus activity (P<0.001, R=0.618).

**Table 3:** Results of E-C4d measurement SLE: systemic lupus erythematosus; E-c4D: erythrocyte-bound complement component 4d; p1: SLE flare-up group vs. SLE without bacterial infection or activity; p2: bacterial infection in the SLE patient group vs. SLE patients without bacterial infection or activity; p3: bacterial infection in the SLE patient group vs. the lupus flare-up group; p4: bacterial infection in the SLE patient group vs. the non-SLE patients with verified bacterial infection group

E-C4d (FI)	SLE flare-up group (N=20)	Bacterial infection in SLE patients (N=20)	Non-SLE patients with culture-proven bacterial infection (N=20)	SLE patients without activity or bacterial infections (N=20)	p-value
Mean ± SD	53.03±14.34	28.97±12.75	22.25±11.10	24.850± 12.10	p1=<0.001
					p2=0.084
					p3=<0.001
					p4=0.301

## Discussion

SLE serves as a model for systemic autoimmune diseases where infection-related mortality is estimated at 25% [14]. Because disease activity presents with fever and malaise, this is often confused with acute infection. The demand for a reliable differentiating tool has become increasingly urgent, particularly in low-income settings. This differentiation helps start antimicrobial therapy in time and possibly reduces the dose of immunosuppression, avoiding many complications for patients and reducing unnecessary expenses.

The aim of this study was to investigate the utility of PCT and E-C4d as early and rapid methods to diagnose and differentiate bacterial infection from flare-up in lupus patients.

The current work studies 60 lupus patients in addition to 20 control subjects. Lupus patients were further subdivided into three groups according to flare-up status, bacterial infection, and lupus with neither flare-up nor bacterial infection.

PCT findings and literature comparison

PCT, a precursor of calcitonin, does not increase in autoimmune diseases [15] and is not affected by glucocorticoids or nonsteroidal anti-inflammatory drugs [16]. In contrast, its production is stimulated by bacterial endotoxins [17].

Previous studies have yielded conflicting results. Some authors have reported elevated PCT levels during infection [18], while others observed no significant difference between bacterial infection and disease flares [19]. In contrast, our findings showed increased PCT levels in patients with confirmed bacterial infections, whereas patients with lupus flares without infection had low PCT levels.

The ROC curve was plotted, and a cutoff of 0.299 ng/ml PCT was found to have 85% sensitivity, 90% specificity, 87.8% PPV, and 95% NPV. Therefore, we suggest that this cutoff may be used to exclude bacterial infection in SLE patients. This high sensitivity and specificity allow recognition of almost all bacterial infections in SLE patients. These findings are similar to the results reported by Yu et al. (2014) [17], who reported a cutoff value of 0.38 ng/ml, with a sensitivity of 74.5% and specificity of 95.5%. Our findings are consistent with those in a previous study conducted on a pediatric cohort with SLE and rheumatoid arthritis in which PCT was used at similar values to distinguish sepsis from flare [20].

In the present study, two patients in the lupus flare-up group had PCT levels above our suggested cutoff value. This might be explained by the possibility of a concurrent infection during the flare-up, which might have been missed by bacteriological cultures.

Role of E-C4d in activity differentiation

A single marker with ideal sensitivity and specificity cannot be recognized as the only diagnostic marker for differentiating between infection and flare-up in SLE patients. This is particularly important in the context of concurrent infection and activity. Therefore, combining more than one marker is highly recommended. The combination should also be able to assist the choice of physicians in increasing the immunosuppressive dose or providing empirical antibiotics without posing a danger to the patient.

Anti-dsDNA antibodies and decreased C3 or C4 levels are well-established markers used routinely to monitor SLE activity [21]. However, the use of C3 and C4 levels to monitor SLE can be unreliable because their hyperconsumption during the active phase of the disease can be offset by inflammation and compensatory hepatic synthesis [22]. Anti-dsDNA is a valuable marker for SLE activity, but is limited by its low sensitivity and its effectiveness as a single reliable diagnostic tool [23].

For these reasons, E-C4d was considered in our study. Several studies have reported elevated levels in lupus flares compared with infection patients [24-26]. Our results revealed significantly elevated levels in lupus patients but not in infection patients, and a positive correlation between the E-C4d score and SLEDAI score for lupus disease activity (p<0.01, R=0.618).

Combined diagnostic utility

The combined use of both serum PCT and E-C4d as a diagnostic utility in lupus patients greatly facilitates differentiating SLE flare-ups from bacterial infections. Serum PCT shows significantly elevated levels in patients with infection, whereas patients experiencing flares showed elevated levels of E-C4d levels. When used together, these two biomarkers may improve the discrimination between the two conditions compared with either marker alone, raising the diagnostic accuracy. However, this conclusion requires validation by a larger study.

Study limitations

The primary limitation of our research was the categorization of patient groups into infected and noninfected groups on the basis of routine aerobic bacterial culture testing. Therefore, there is a potential for false-negative culture results. In addition, we cannot exclude viral infections in our patients as one of the potential causes of elevated PCT levels. In addition, the conclusion of using the combined marker to raise diagnostic differentiation of infection from lupus activity requires a larger prospective study.

## Conclusions

Differentiating between bacterial infection and disease flare in patients with SLE remains a critical clinical challenge, particularly in resource-limited settings. Our findings support the utility of PCT as a sensitive and specific biomarker for identifying bacterial infections in lupus patients, with a suggested cutoff of 298.66 pg/ml offering strong diagnostic performance. Additionally, E-C4d demonstrated a significant correlation with lupus disease activity and may serve as a complementary marker in distinguishing flare from infection. A combined approach using PCT and E-C4d might be suggested as a tool to improve clinical decision-making regarding immunosuppressive therapy and antimicrobial use. Further research with broader pathogen detection and larger patient cohorts is recommended to validate these findings and refine diagnostic strategies before implementation in the general population.
